# Uncovering the therapeutic potential of anti-tuberculoid agent Isoniazid in a model of microbial-driven Crohn’s disease

**DOI:** 10.1093/ecco-jcc/jjaf032

**Published:** 2025-02-23

**Authors:** Matthew Stephens, Keith Keane, Simon Roizes, Manon Defaye, Christophe Altier, Pierre-Yves von der Weid

**Affiliations:** Department of Physiology and Pharmacology, Cumming School of Medicine, University of Calgary, Calgary, Alberta T2N4N1, Canada; Inflammation Research Network Snyder Institute for Chronic Diseases, Cumming School of Medicine, University of Calgary, Calgary, Alberta T2N4N1, Canada; Snyder Institute for Chronic Diseases, University of Calgary, HS 1665, 3330 Hospital Drive NW, Calgary, Alberta T2N4N1, Canada; Department of Physiology and Pharmacology, Cumming School of Medicine, University of Calgary, Calgary, Alberta T2N4N1, Canada; Inflammation Research Network Snyder Institute for Chronic Diseases, Cumming School of Medicine, University of Calgary, Calgary, Alberta T2N4N1, Canada; Snyder Institute for Chronic Diseases, University of Calgary, HS 1665, 3330 Hospital Drive NW, Calgary, Alberta T2N4N1, Canada; Department of Physiology and Pharmacology, Cumming School of Medicine, University of Calgary, Calgary, Alberta T2N4N1, Canada; Inflammation Research Network Snyder Institute for Chronic Diseases, Cumming School of Medicine, University of Calgary, Calgary, Alberta T2N4N1, Canada; Snyder Institute for Chronic Diseases, University of Calgary, HS 1665, 3330 Hospital Drive NW, Calgary, Alberta T2N4N1, Canada; Department of Physiology and Pharmacology, Cumming School of Medicine, University of Calgary, Calgary, Alberta T2N4N1, Canada; Inflammation Research Network Snyder Institute for Chronic Diseases, Cumming School of Medicine, University of Calgary, Calgary, Alberta T2N4N1, Canada; Snyder Institute for Chronic Diseases, University of Calgary, HS 1665, 3330 Hospital Drive NW, Calgary, Alberta T2N4N1, Canada; Hotchkiss Brain Institute, Cumming School of Medicine, University of Calgary, Calgary, Alberta T2N4N1, Canada; Department of Physiology and Pharmacology, Cumming School of Medicine, University of Calgary, Calgary, Alberta T2N4N1, Canada; Inflammation Research Network Snyder Institute for Chronic Diseases, Cumming School of Medicine, University of Calgary, Calgary, Alberta T2N4N1, Canada; Snyder Institute for Chronic Diseases, University of Calgary, HS 1665, 3330 Hospital Drive NW, Calgary, Alberta T2N4N1, Canada; Hotchkiss Brain Institute, Cumming School of Medicine, University of Calgary, Calgary, Alberta T2N4N1, Canada; Department of Physiology and Pharmacology, Cumming School of Medicine, University of Calgary, Calgary, Alberta T2N4N1, Canada; Inflammation Research Network Snyder Institute for Chronic Diseases, Cumming School of Medicine, University of Calgary, Calgary, Alberta T2N4N1, Canada; Snyder Institute for Chronic Diseases, University of Calgary, HS 1665, 3330 Hospital Drive NW, Calgary, Alberta T2N4N1, Canada

**Keywords:** inflammatory bowel disease, Isoniazid, pathobiont, therapeutics, pathophysiology

## Abstract

**Aims:**

TNFα has long stood as a hallmark feature of both inflammatory bowel disease and arthritis with its therapeutic potential demonstrated in neutralizing monoclonal antibody treatments such as Infliximab. Due to the high global burden of latent *Mycobacterium tuberculosis* (TB) infections, prior to receiving anti-TNF therapy, patients testing positive for latent TB are given prophylactic treatment with anti-tuberculoid medications including the first described TB-selective antibiotic, Isoniazid. While this is common clinical practice to prevent the emergence of TB, little is known about whether Isoniazid modifies intestinal inflammation alone. The aim of this study, therefore, was to determine whether Isoniazid presents a novel TB-independent therapeutic option for the treatment of Crohn’s disease (CD)-like ileitis and uncover new mechanisms predisposing the host to intestinal inflammation.

**Methods:**

The transgenic TNF^ΔARE^ mouse model of Crohn’s-like terminal ileitis was used. The impact of Isoniazid administration (10 mg/kg/day dose in drinking water) on disease development was monitored between 8 and 12 weeks of age using a variety of behavioral and serological assays. Behavioral and motor functions were assessed using the LABORAS automated monitoring system while systemic and local tissue inflammation were determined at experimental termination using multiplex cytokine analysis. Whole-mount tissue immunofluorescence and fluorescent in situ hybridization were used to qualify changes within the host as well as the microbial compartment of the ileum and associated mesentery. Proposed cellular mechanisms of altered cytokine decay were performed on isolated primary splenocytes in vitro using selective pharmacological agents.

**Results:**

Compared to age-matched wild-type littermates, TNF^ΔARE^ mice display prominent progressive sickness behaviors from 8 through 12 weeks of age indicated by reduced movement, climbing, and rearing. Prophylactic administration of Isoniazid (10 mg/kg/day) is effectively able to protect TNF^ΔARE^ mice from this loss of function during the same period. Analysis revealed that Isoniazid was able to significantly reduce both systemic and intestinal inflammation compared to untreated vehicle controls impacting the epithelial colonization of known pathobiont segmented filamentous bacteria (SFB). Reduction in terminal ileal inflammation was also associated to the diminished formation of precursor-tertiary lymphoid organs within the associated ileal mesentery which were found to be associated with endospores derived SFB itself. Finally, we reveal that due to their genetic manipulation, TNF^ΔARE^ mice display accelerated posttranscriptional decay of IL-22 mRNA resulting in diminished IL-22 protein production and associated downstream antimicrobial peptide production.

**Conclusions:**

Isoniazid protects against the development of intestinal and systemic inflammation in the TNF^ΔARE^ model of terminal ileitis by limiting the expansion of mucosal SFB and progression of the associated microbial-driven inflammation. This work highlights a possible mycobacterial-independent function of Isoniazid in limiting CD pathophysiology through limiting the mucosal establishment of pathobionts such as SFB and the association of such microbe-derived endospores linked to the formation of ectopic tertiary lymphoid organs seen commonly in patients.

## Graphical Abstract

**Graphical Abstract:**
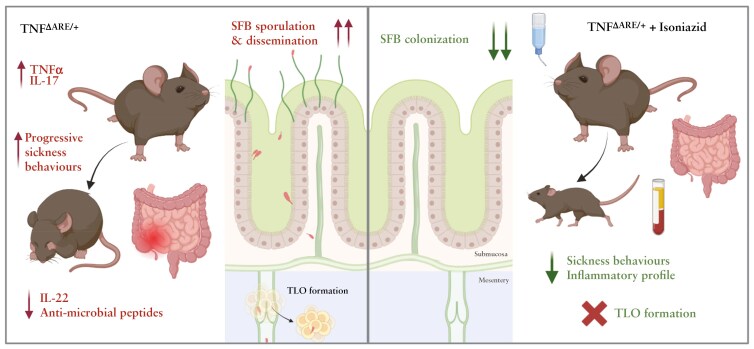

## 1. Introduction

Inflammatory bowel disease (IBD) is estimated to effect almost 1 million people in North America and projected to increase over the years to come.^1,2^ Divided into 2 groups, ulcerative colitis (UC) and Crohn’s disease (CD), disease etiology remains incomplete and thus treatment options for IBD are limited. One revolutionary drug class used in the treatment of IBD is monoclonal antibodies such as Infliximab which, through targeted sequestration of the pro-inflammatory cytokine TNFα, has effectively reduced disease burden in numerous patients. However, a clinical concern when treating patients with such immunosuppressive therapies is the emergence of latent infectious diseases epitomized by *Mycobacterium tuberculosis* (TB).^3^ It is predicted that over ¼ of the global population is infected with TB but, despite its prevalence, a majority of these individuals do not progress due to active tuberculosis and are instead able to contain the bacterial infection developing an asymptomatic, latent form of the disease. As such, the number of latent TB cases globally could be markedly higher than predicted.^4^ Many of the estimated 2.3 billion people infected with latent TB are at risk for developing active disease, especially immunocompromised individuals, such as those with HIV coinfections,^5,6^ or those on immunosuppressive medications.^7^ While commercially available testing for latent TB is available, many fail to determine whether an individual has cleared the infection or harbors persistent bacilli reservoirs.^8^ Due to the necessity of TNFα signaling in the maintained quiescence of latent tuberculoid granulomas, anti-TNF therapy use is carefully weighted with prophylactic treatments given to minimize adverse outcomes.^9,10^

Isoniazid, given daily for 6-9 months, continues to be the most widely prescribed tuberculosis preventative therapy globally due to its equitable accessibility, good tolerance, and evidence of effectiveness (2020 WHO TPT guidance). Originally synthesized in 1912, Isoniazid was described as a highly selective pro-antibiotic, requiring microbial processing via the TB-specific enzyme catalase-peroxidase coded by the gene KatG, which catalyzes Isoniazid into a range of chemically reactive intermediates, including isonicotinoyl radicals.^11,12^ The KatG-dependent mechanism initially described therefore allowed for selective targeting of KatG^+^ bacilli, inhibiting mycolic acid cell wall synthesis,^13^ an essential component of mycobacillin replication. Interestingly, Isoniazid processing is not solely limited to mycobacteria as many microbes, including the “lab workhorse” *Escherichia coli* K12, contain functional KatG albeit are not sensitive to the antimicrobial effects due to lack of a mycolic acid cell wall.^14^

Almost 25 years ago, George Kollias and his team developed one of the most human-like terminal ileitis mouse models that has become a staple for the research community.^15^ Through deletion of the AU-rich 3′-ARE domain of the TNF gene locus, these mice contain stable TNF mRNA transcripts that are protected from posttranscriptional RNA-binding deadenylases such as tristetraprolin (TTP).^16,17^ The hemizygous model TNF^ΔARE/+^ (herein referred to as TNF^ΔARE^) displays stable and progressive development of intestinal and joint pathology similar to clinical manifestations of CD and rheumatoid arthritis with spondylarthritis.^15,18–20^ In the initial publication, loosely formed noncaseating granulomas were described to be present within the ileum of inflamed mice, but it was not until almost 2 decades later that we^19^ and others^20^ described the existence of extraintestinal lymphoid aggregates, termed tertiary lymphoid organs (TLOs). What drives the formation of granulomatous TLO structures, whether they are protective or deleterious to disease, and the context in which they arise has so far only been speculated, appearing to be unique in function as has been described in other TLO-associated diseases.^21^ What is known, however, it that neutralization of TNFα with Infliximab is effectively able to diminish the formation of these TLOs within the TNF^ΔARE^ model of CD-like ileitis, loosely suggesting they are produced in part formed or regulated by TNFα itself.^20^ After our own determination of the composition of the TNF^ΔARE^ mesenteric TLO, we noted it to be highly similar to that of a granuloma consisting of a composite of: macrophage, T and B cells, and occasionally other immune cells including neutrophils and eosinophils.^19^ While both granulomas and lymphoid tissues share common cellular compositions, the purpose and development of each are unique but with their high prevalence in patients of chronic inflammatory diseases, their etiology and function still need to be determined.

Works published simultaneously by Schaubeck and Roulis in 2016 suggested the TNF^ΔARE^ inflammatory phenotype is essentially driven by the microbiota as, through germ-free (GF) derivation of the strain, systemic and local inflammation was abrogated.^22,23^ Furthermore, both groups outlined the presence of epithelial–adherent filamentous bacteria of unknown origin. A recent follow-up study led by Metwaly from Dr. Haller’s group highlighted the crucial role of segmented filamentous bacteria (SFB), *Candidatus arthromitus*, in driving ileo-colonic inflammation. Their findings showed that rederiving germ-free (GF) mice to specific pathogen-free (SPF) conditions—without the presence of SFB—failed to induce the same inflammation observed in their colony, emphasizing SFB’s essential role in this process.^24^ SFB is a commensal microbe that attaches directly to the ileal epithelium and potently stimulates a host Th17 immune response but in an immunocompetent host, its presence is not pathological with its adherence and shedding from the epithelium regulated by rhythmic production of antimicrobial peptides (AMPs) such as Reg3γ.^25^ Why SFB is pathological in the TNF^ΔARE^ mice seems to be a combination of SFB, genetics, and other members of the intestinal microbiome, yet whether similar pathobionts exist in patient cohorts remains inconclusive.

Taken together, these previous findings led us to question whether the contentious literature of mycobacteria as a driver of IBD pathology may be in fact a “red-herring” caused by our own assumptions regarding antibiotic specificity and overall refined antimicrobial function. Isoniazid is thought to be a mycobacterial selective antibiotic but has shown numerous unrelated impacts on other microbes as well as the host. Therefore, we speculated that other gram-positive microbes, such as the recently identified pathobiont SFB, may be susceptible to Isoniazid or that the host’s inflammatory environment may be calmed by the host-directed functions of Isoniazid.

## 2. Materials and methods

### 2.1. Ethical approval and mouse use

All mice were housed at a constant temperature (22 °C) and maintained on a 12:12-hour light-dark cycle, with food and water ad libitum. Mice were assessed at 8 and 12 weeks (±2 days) of age with experiments replicated 3-4 separate times within each treatment group to ensure reproducibility. Animal handling and associated experiments were approved by the University of Calgary Animal Care and Ethics Committee and conformed to the guidelines established by the Canadian Council of Animal Care (Protocol Number AC20-0051).

### 2.2. Genotyping of mice

Genomic DNA was isolated from 3-mm ear punches of mice at 3-4 weeks of age. Samples were processed using the Extract-N-Amp tissue PCR kit (Sigma-Aldrich, Cat. No. XNAT2) according to the manufacturer’s instructions. DNA isolated from this extraction was used as the template for the genotyping PCR. Briefly, 4 µL of DNA template was added to 16 µL of premixed PCR master mix consisting of 10 µL 2× PCR master mix (Promega, Cat. No. M7502) and 0.5 µL of each TNF-specific primer (F′ AATGCACAGCCTTCCTCACAGAG, R′ AATTAGGGTTAGGCTCCTGTTTCC) designed to capture the entire 3′UTR of the TNF transcript and optimized allowing accurate genotyping to be performed in <3 hours. Five microlitres of nuclease-free water were added to bring the final reaction volume to 20 µL. Samples were mixed briefly to ensure even distribution of reagents. The PCR reaction was performed on a Biorad T100 thermocycler under the following conditions: initial denature step—95 °C for 1 minute, followed by 34 cycles of: 95 °C for 30 seconds, 58 °C for 20 seconds, and 68 °C for 40 seconds. A final elongation step at 68 °C following the final cycle before the block is chilled at 4 °C. A 2.5% TAE agarose gel was used to confirm band size with wild type (WT) showing a single band at 465 bp and TNF^ΔARE/+^ mice showing 2 distinct bands, one at 465 bp confirming the WT allele and one at ~533 bp which corresponds to the 68-bp insertion mutation previously documented within the TNF^ΔARE^ mice.^15^

### 2.3. Laboratory Animal Behavior Observational, Registration and Analysis System

Sickness behaviors of the mice were assessed during nocturnal activity using the noninvasive rodent behavior recognition system Laboratory Animal Behavior Observational, Registration and Analysis System (LABORAS; Metris). LABORAS is a fully automated behavior recognition and tracking system using mechanical vibrations to classify different natural behaviors (eg, eating, drinking, climbing, rearing, locomotion, immobility, etc.) and has previously been validated for pharmacological studies.^26^ Mice were placed in the LABORAS cages for a period of 16 hours (5:00 pm to 9:00 am), with drinking water and food available ad libitum and under normal light cycles.

### 2.4. Isoniazid administration

Isoniazid was dissolved in autoclaved drinking water at a concentration of 25 mg/L to give a concentration of 25 µg/mL. Mice at 8 weeks of age were monitored and observed to drink an average of 4.7 ± 0.8 mL water per day translating to a dose of 97.5-137.5 µg Isoniazid per mouse per day (~10 mg/kg/day). Variation in mouse weight was limited by excluding the use of cage alpha males which were defined to exceed 10% extra body mass compared to their cage mates to ensure correct dosing.

### 2.5. Whole-mount immunofluorescence staining and imaging

The protocol used for immunofluorescent staining of the mesentery has been described previously.^19,27,28^ Briefly, terminal ileal mesenteric arcades were dissected from externalized mouse intestines, pinned in sylgard-coated 35-mm dishes, and fixed in 10% formalin for 1-2 hours at room temperature on an orbital shaker. Samples were then washed 3 times in PBS before being transferred into PBS containing 0.1% sodium azide for prolonged storage (not to exceed 2 weeks at 4 °C). Mesenteric whole mounts were blocked with 2% BSA in PBST (0.3% Triton X-100) for 1 hour at RT before washing and then incubating overnight with 0.5 µg/mL anti-mouse CD45 (BioLegend, Cat. No. 103102) antibody at 4 °C. Samples were then washed 3 times for 10 minutes in PBST (0.3% Triton X-100) before being probed with a 5 µg/mL of Chicken anti-Rat Alexa488 conjugated secondary antibody (Invitrogen, Cat. No. A48269) alongside Cy3 primary conjugated and 0.5 µg/mL Anti-Actin α-Smooth Muscle (Sigma-Aldrich, Cat. No. C6198) for 2 hours at RT under orbital agitation alongside 0.1 µg/mL DAPI. After staining, samples were washed 3 times in PBST (0.3% Triton X-100) for 15 minutes before being sequentially dehydrated in ethanol (50%, 70%, 90%, 100%) for 5 minutes at each concentration. During this dehydration step the samples become much more translucent. Dehydrated whole-mount samples were then transferred onto a charged super frost slide before being treated with 100-200 µL methyl salicylate (Fisher Scientific, Cat. No. M-220) causing rapid optical clearing. A coverslip was added and fixed in place with clear nail polish. Example images generated with antibody Isotype controls can be found and detailed in Supplementary Figure S1. All images were taken on an inverted Leica SP8 confocal microscope and analyzed using Leica LASX software (Leica Microsystems Canada Inc.).

### 2.6. Fluorescent in situ hybridization

Intestinal samples (1-2 cm lengths) were flushed with PBS before being fixed in 10% formalin for 16 hours at 4 °C. Samples were rinsed briefly with 1× PBS before being transferred into a 30% sucrose solution for 24 hours or until the sample had sunk completely (whichever took longer). Sucrose-fixed samples were cut into 4-mm sections using a clean scalpel and then imbedded in OCT within a cryomold and snap-frozen at −80 °C. Samples were sectioned to 10 µm using a cryostat and stored at −80 °C until staining was performed. Bacteria were permeabilized using 40 mg/mL Lysosome (GoldBio, Cat. No. L-040-1) reconstituted in fluorescent in situ hybridization (FISH) buffer (2 mM EDTA, 2 mM TRIS, 1.2% Triton X-100) for 45 minutes at 37 °C in a humidified chamber. After samples were permeabilized, slides were rinsed with 1ml of 1× PBS and stained with microbial FISH probes (Eub338 probe (pan-eubacteria) – /5AF647/GCTGCCTCCCGTAGGAGT-3′ SFB probe (murine *C. arthromitus*) – /5Cy3/GCGAGCTTCCCTCATTACAAGG) at a final concentration of 100nM in FISH buffer at 46 °C O/N in a humidified chamber. Samples were washed thoroughly with 1× PBS before being counterstained with DAPI and mounted with a coverslip and anti-fade mounting solution (Vector Laboratories, Cat. No. PC2095933).

### 2.7. Ileal H&E staining and gross-structural measurements

Previously processed and sectioned samples were stained using the following H&E staining protocol with incubation times modified for cryosection samples. Water—8 minutes, Gill II Hematoxylin—7 minutes, Water—15 seconds, acidified 100% Ethanol—30 seconds, Scott’s Blueing solution—30 seconds, Water—1 minute, Eosin—2.5 minutes, Water—10 seconds, 70% Ethanol—1 minute, 90% Ethanol—1 minute, 95% Ethanol—1 minute, 100% Ethanol—1 minute, 100% Ethanol—1 minute, Neoclear—2 minutes, Neoclear—4 minutes, let completely dry then mount in aqueous mounting media (Glycerin). Samples were imaged on a Leica DM2700M upright microscope. Villus–crypt length, crypt width at midpoint, crypt depth, and muscle layer thickness were measured. Cellular infiltration (score 0-3), mucosal alteration (score 0-3 [vasculitis, muscular thickening, and crypt hyperplasia]), and submucosal edema (absent, moderate, and severe) were analyzed and totaled into an inflammatory index using Leica LASX office software in a randomized manner by 3 sample-blinded investigators.

### 2.8. Flow cytometry

Single cells from luminally flushed ileal samples were collected and isolated as previously described.^27,28^ Briefly, 0.5-1 cm lengths of the terminal ileum (free of peyers patches) were flushed gently of their luminal contents with sterile 1× DPBS (Sigma-Aldrich, Cat. No. D8537). Ileal sections were then finely minced with sterile scissors and transferred into an enzymatic digestion cocktail (RPMI + 10 mg/mL Collagenase I, 1 mg/mL DNAse I) for 30 minutes in a 37 °C water bath with gentle agitation every 10 minutes. Digested cells were passed through a 70-µm strainer to create a single-cell suspension and then washed in FACS buffer (DPBS + 0.5% BSA and 0.1% NaN_3_). Purified cells were blocked using anti-CD16/32 antibody for 5 minutes at room temperature and then stained with surface antibodies mCD45 (Biolegend, Cat. No. 103129) and mCD4 (Biolegend, Cat. No. 100421) on ice and in the dark for 30 minutes. After washing with FACS buffer, the cells were then stained for intracellular makers FOXP3 and RORγt using the eBioscience Transcription Factor Staining Buffer Set according to the manufacturer’s guidelines. Briefly, after surface staining samples were fixed in 100 µL of 1× Fixation Buffer for 30 minutes at room temperature in the dark. Two milliliters of 1× Permeabilization buffer were added and cells pelleted at 600 g for 5 minutes. This step was repeated again to remove any residual permeabilization buffer. Cells were then resuspended in 100 µL of 1× Permeabilization buffer and FOXP3 (BD Bioscience, Cat. No. 562996) and RORγt (BD Bioscience, Cat. No. 562607) primary conjugated antibodies used at 1:20 dilution (100 µg/mL) and incubated in the dark for 60 minutes. Cells were washed in 1× Permeabilization buffer and resuspended in 400 µL FACS buffer prior to analysis. Data were acquired using a BD FACSCanto and data analyzed using FlowJo10.10.

### 2.9. Multiplex serum and tissue cytokine array

Whole blood was collected from mice without any preservatives or anticoagulants and left at room temperature for 10-20 minutes to allow for sufficient clotting. Serum was separated by bench-top centrifugation 2300*g* for 10 minutes. Samples that were hemolyzed were not used for analysis and discarded. Terminal ileal samples were surgically isolated, cleaned of luminal content with sterile PBS, and dabbed with Kimwipes to remove excess moisture. Samples were resuspended in 10× weight/volume Tris-HCL lysis buffer containing 100 mM NaCl, 200 mM Tris-HCL (pH 8), 1% Triton X-100, 0.5% SDS, 1 mM EDTA, 1 mM Na_3_VO_4_, and 25 mM NaF. Protease inhibitor cocktail (Cell Signalling Technology, Cat. No. 5871, 100×) was also added to a final concentration of 1×. Processed samples were analyzed by Eve Technologies using their mouse 32 plex discovery assay (Cat. No. MD32) at a 1:2 dilution.

### 2.10. SFB quantification in tissue and feces

Fecal pellets extracted from the carcass of freshly deceased mice were collected in a sterile 1.5-mL Eppendorf and snap-frozen at −80 °C for downstream processing. DNA extraction and purification from fecal pellets were performed using Power Fecal Pro DNA extraction kit according to the manufacturer’s instructions (Qiagen, Cat. No. 51804). Isolation of DNA was performed using a similar methodology from luminally flushed intestinal samples with the addition of a mechanical homogenization step. Quantification was assessed using SensiFAST SYBR Mastermix (Bioline, Cat. No. BIO-92005) and specific primer pair for mSFB (F′ GACGCTGAGGCATGAGAGCAT, R′ GACGGCACGGATTGTTATTCA)^29^ as well as nonselective primers amplifying bacterial 16S ribosomal RNA gene (F′ TGTGGGTTGTGAATAACAAT, R′ GCGGGCTTCCCTCATTACAAGG), here used as an internal reference to normalize the amount of input DNA within each sample.^30^ One hundred and fifty nanograms of input DNA were used and gene amplification conducted using PCR primers with the amount of template adjusted to standardize the amount of 16S product amplified. A Ct >30 was determined as nonspecific or negative due to the production of nonspecific amplicons produced at 32-37 cycles. The amplified SFB and 16S PCR products were confirmed on 2% TAE agarose gels.

### 2.11. Myeloperoxidase activity assay

Myeloperoxidase (MPO) activity of ileal tissue was determined as previously described.^19,28^ Ileum was surgically isolated and flushed of luminal content by briefly washing in sterile PBS. The sample was dabbed dry on a tissue before being weighed. The sample was then homogenized in 40× w/v HTAB buffer (5g hexadecyltrimethylammonium bromide in 1 L potassium phosphate buffer [Solution A: 6.8 g monobasic potassium phosphate in 1 L dH_2_O + Solution B: 8.7 g dibasic potassium phosphate in 1 L dH_2_O final pH 6.0]) in a bullet blender. Samples were clarified by centrifugation at 13 000*g* for 10 minutes and MPO activity measured against a standard curve of human MPO (100 U/mL, Calbiochem, Cat. No. 475911). The rate of activity was determined by microplate spectrophotometry quantifying the colored substrate produced by MPO interacting with O-dianisidine (Sigma-Aldrich, Cat. No. D3252) and 1% hydrogen peroxide. Absorbance value and thus calculated MPO activity were normalized to the weight of the tissue input.

### 2.12. Splenocyte stimulation for mRNA decay and cytokine production assays

Primary isolated splenocytes were isolated from freshly euthanized WT and TNF^ΔARE^ mice through gentle disruption of the intact isolated spleen through a 100-µm cell strainer with the end of a sterile 1-mL syringe plunger. Single cells were washed with sterile 1× DPBS (Sigma-Aldrich, Cat. No. D8537) and pelleted before red blood cells were lysed in 2 mL/spleen of ACK lysing buffer (ThermoFisher, Cat. No. A1049201) for 10 minutes at room temperature. Cells were washed again, this time in complete RPMI (+10% heat-inactivated FCS + 1% Pen/Strep) and counted using a hemacytometer with trypan blue counterstaining to determine cell viability. 1 × 10^6^ live splenocytes were plated in each well of a 12-well tissue culture plate and left overnight in a cell culture incubator (37 °C, 5% CO_2_) to equilibrate. The following morning cells were stimulated with PMA (50 ng/mL) and Ionomycin (1 µg/mL) in fresh pre-warmed complete RPMI for 1 hour in the cell culture incubator. To halt transcriptional activity, Actinomycin-D (5 µg/mL) or vehicle control (DMSO 0.05% v/v) was added after the 1-hour stimulation period and samples were harvested at 0 hour, 1 hour, 2 hours, and 4 hours post-Actinomycin-D administration. Supernatants from matched samples stimulated with PMA/ionomycin, but without Actinomycin-D treatment, were collected to determine cytokine production after 6 and 24 hours. To inhibit deadenylase activity Neomycin-B (Sigma-Aldrich, Cat. No. 1458019) was administered at 10 µg/mL during PMA/Ionomycin stimulation and maintained in solution throughout the experiment.

### 2.13. RNA isolation and splenocyte mRNA decay assay

At the predefined time points, total RNA was isolated from splenocytes using the RNeasy column-based cleanup kit according to the manufacturer’s instructions (Qiagen). cDNA conversion was performed using the All-in-One 5× RT MasterMix (Cat. No. G592, Applied Biological Materials), GAPDH (Forward 5′-CATCACTGCCACCCAGAAGACTG-3′, Reverse 5′-ATGCCAGTGAGCTTCCCGTTCAG-3′) was used as the housekeeping gene, and IL-22 mRNA (Forward: 5′-GCTTGAGGTGTCCAACTTCCAG-3′, Reverse: 5′-ACTCCTCGGAACAGTTTCTCCC-3′) was assessed via qPCR using the BlasTaq 2× qPCR MasterMix (Cat. No. G891, Applied Biological Materials). Relative changes in mRNA expression compared to unstimulated controls and normalized to GAPDH were quantified using the 2^−ΔΔCt^ method or represented as % maximal induction compared to the abundance of IL-22 after 1 hour of PMA/Ionomycin stimulation (as used in Figure 8). AMPs: Reg3β (Forward 5′-TGGCTCCTACTGCTATGCCTTG-3′, Reverse 5′-CGCTATTGAGCACAGATACGAGG-3′), Reg3γ (Forward 5′-CGTGCCTATGGCTCCTATTGCT-3′, Reverse 5′-TTCAGCGCCACTGAGCACAGAC-3′), and S100A9 (Forward 5′-TGGTGGAAGCACAGTTGGCAAC-3′, Reverse 5′-CAGCATCATACACTCCTCAAAGC-3′) were quantified from total terminal ileal homogenates normalizing values to that of WT vehicle controls (as seen in Figure 7).

### 2.14. Enzyme-linked immunosorbent assays

IL-22 produced by splenocytes was measured using the ELISA MAX^TM^ Deluxe Set Mouse IL-22 (Cat. No. 436304, Biolegend) as per manufacturer’s guidelines with supernatants remaining undiluted. Absorbance values were measured using a SpectraMax i3 spectrophotometer and concentrations calculated against the standard curve using Microsoft Excel.

### 2.15. Statistical analysis

The data and statistical analysis were designed to generate treatment groups of equal size with randomized conditions separated into at least 3 independent experiments with blinded coinvestigator reinforced analysis. Group sizes represent the number of independent values (biological replicates) with statistical analysis performed only on these values which are a calculated average of repeated technical replicates. Where indicated, variable or arbitrary values were normalized (correction of test values to baseline or control group), using fold-matched control values. All data are expressed as the mean ± standard error of the mean (SEM) unless indicated otherwise. Normality of data was assessed on all data sets with parametric data analyzed by 2-tailed Student’s *t*-tests and nonparametric data analyzed using Mann–Whitney *U* tests or ANOVA with appropriate post hoc tests as indicated. Data were considered significant with a *P*-value of less than .05 with all statistics performed using GraphPad 9 software (GraphPad Software Inc., GraphPad Prism, RRID: SCR_002798).

## 3. Results

### 3.1. Noninvasive behavioral monitoring demonstrates that Isoniazid is effective in limiting the progression of sickness behavior of the TNF^ΔARE^ model of terminal ileitis

**Figure 1.**
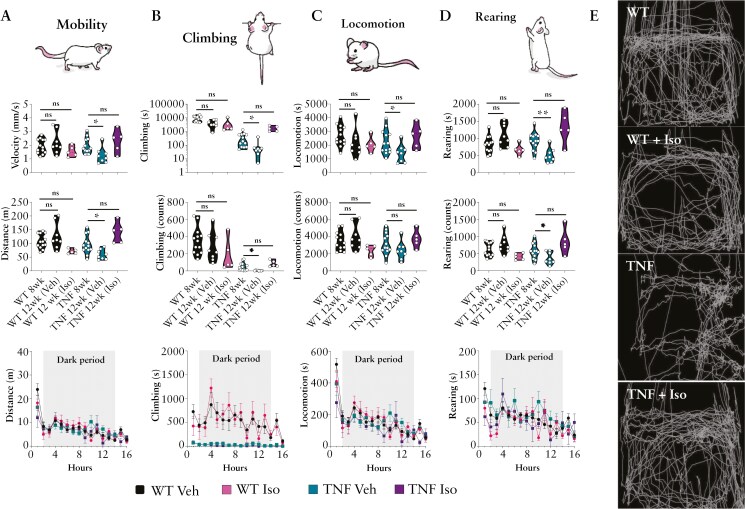
Sickness and disease behavior in the progression of TNF^ΔARE^ mice during Isoniazid prophylaxis. Mice were administered Isoniazid in drinking water between 8 and 12 weeks of age. Biomechanical and behavioral metrics were measured using a LABORAS system and monitored during the active (dark–light cycle) period. (A) Metrics of mobility including average velocity, total distance traveled, (B) climbing duration and frequency, (C) locomotion duration and frequency, and (D) rearing duration and frequency were measured across a 16-hour time period. (E) Representative movement traces of an individual mouse across a 30-minute period. Data are represented as mean with truncated violin plots encapsulating separate values gathered from *n* = 5 mice per treatment group with experiments performed across 3 or more separate occasions. Statistical analyses were performed using a 1-way ANOVA with Tukey’s post hoc test comparing pretreatment (8 weeks) to posttreatment values (12-week-old). *P < .05, **P < .01.

Abdominal pain is a primary symptom of IBD, with 40%-90% of patients experiencing it during acute intestinal inflammation. Within the GI tract, inflammatory mediators produced by both infiltrating and resident immune cells have been shown to drive neuronal sensitization, resulting in visceral pain. Measuring visceromotor responses to colorectal distention can provide valuable insights into visceral pain in these mice. However, their colons are fragile, making the risk of perforation especially high. Therefore, consistent with studies examining pain in pancreatic and colitis models,^31–33^ we assessed changes in sickness and intestinal inflammation using the LABORAS system which monitored behaviors associated with visceral discomfort and pain. We monitored the distance traveled by the animals as well as the amount of time they spent climbing and rearing, activities which involve stretching the abdominal muscles, making them susceptible to the effects of visceral pain associated with intestinal inflammation. Therefore, at 8 weeks of age, TNF^ΔARE^, along with age and litter-matched WT littermate counterparts, were assessed for baseline sickness behavioral and physical characteristics indicative of the intestinal discomfort (rearing), systemic inflammation and lethargy (mobility and locomotion), and associated forelimb arthritis (climbing), before being administered Isoniazid (10 mg/kg/day) contained within their drinking water for 4 weeks (Figure 1A). Water consumption was monitored at the time of experimentation to confirm correct dosing. If untreated, TNF^ΔARE^ mice display a significant loss in multiple motor functions indicative of progressive sickness behavior, arthritis, and abdominal pain including reduced velocity and distance traveled (Figure 1A), climbing duration and frequency (Figure 1B), locomotion duration and frequency (Figure 1C), and rearing duration and frequency (Figure 1D). TNF^ΔARE^ mice treated with Isoniazid (TNF Iso) were protected from these changes, maintaining functions similar to WT vehicle controls (WT Veh) or at least, their 8-week-old baseline values (Figure 1A–D). A representative trace from randomly selected mice from each group at the termination of the experiment shows the movement of a single mouse across a 10-minute period (Figure 1E) highlighting the altered movement patterns of WT and TNF^ΔARE^ mice and the impact of Isoniazid treatment. While these observational data show promise for the usefulness of the LABORAS system for monitoring the progression of this disease model, it remains open to interpretation whether these behavioral changes can be definitively assigned to individual pathologies, are a direct result of pain, or are influenced by overall fatigue or ill-health.

### 3.2. Isoniazid prophylaxis limits the progression of several disease metrics

**Figure 2.**
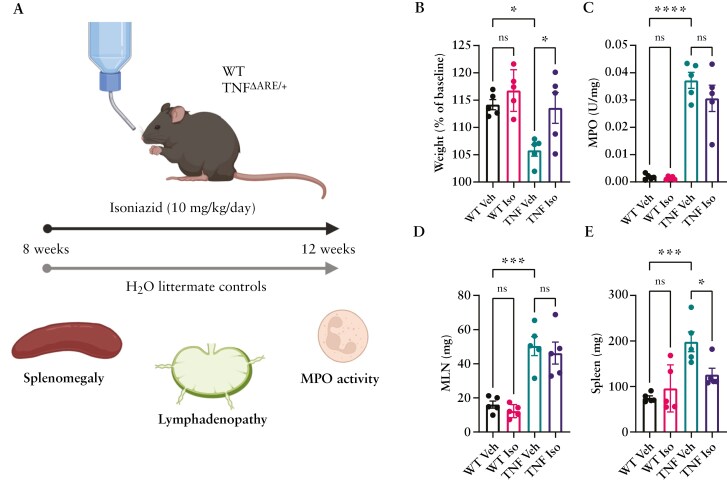
Impact of Isoniazid administration on the progression of TNF^ΔARE^ associated pathophysiology. (A) Schematic of Isoniazid treatment regime. (B) % weight change of mice at 12 weeks of age, in relation to their baseline weight taken at 8 weeks of age. Impact of Isoniazid treatment on (C) ileal myeloperoxidase (MPO), (D) MLN, or (E) spleen weight of WT and TNF mice at 12 weeks of age with or without Isoniazid supplementation (10 mg/kg/day). Data are represented as mean ± SEM of *n* = 5 mice per group. Statistical analyses were performed using a 1-way ANOVA with Tukey’s post hoc test. *P < .05, **P < .01, ****P < .0001.

TNF^ΔARE^ mice display multiple human-like CD-associated pathologies including failure to gain weight,^34^ polyarthritis^35^, and spondylarthritis^36^ beginning at 4-6 weeks of age, thought to be driven by the concurrent development of transmural, microbiota-dependent terminal ileitis.^18–20,22^ With our own works documenting the progressive ileal inflammation^37–39^ within the TNF^ΔARE^ and other models of IBD,^19,27^ we sought to assess the impact of Isoniazid treatment on these disease metrics at the end of 4 weeks of treatment (treatment regime depicted in Figure 2A). In line with previous findings, compared to WT counterparts, TNF^ΔARE^ mice gain significantly less weight between 8 and 12 weeks of age (Figure 2B). WT vehicle (water only) mice gained on average 14.16 ± 2.47% body mass over the 4-week period with similar values found with WT mice treated with Isoniazid (14.76 ± 7.11%). TNF^ΔARE^ mice, however, only gain on average 6.06 ± 2.96% body mass within the same time period. This reduced weight gain was, however, significantly improved in TNF^ΔARE^ mice which received Isoniazid (13.58 ± 7.47%). Gross intestinal inflammation, assessed by MPO activity, was elevated within the ileum of vehicle TNF^ΔARE^ mice (*P* < .0001) and was unchanged with Isoniazid administration (Figure 2C). Mesenteric lymph node (MLN) lymphadenopathy, found to be significantly increased in TNF^ΔARE^ mice (49.43 ± 15.53 mg) compared to WT controls (16.06 ± 4.76 mg), was also not impacted by Isoniazid treatment (46.22 ± 10.22 mg) (Figure 2D). Spleen weight (splenomegaly), indicative of systemic inflammation, was increased in vehicle-treated TNF^ΔARE^ compared to WT mice (*P* < .001) (192.76 ± 71.96 mg vs 88.43 ± 7.84 mg, respectively) and was significantly reduced with Isoniazid treatment (*P* < .05, 125.95 ± 52.05 mg, Figure 2E).

### 3.3. Systemic inflammation is reduced during prophylactic Isoniazid treatment

**Figure 3.**
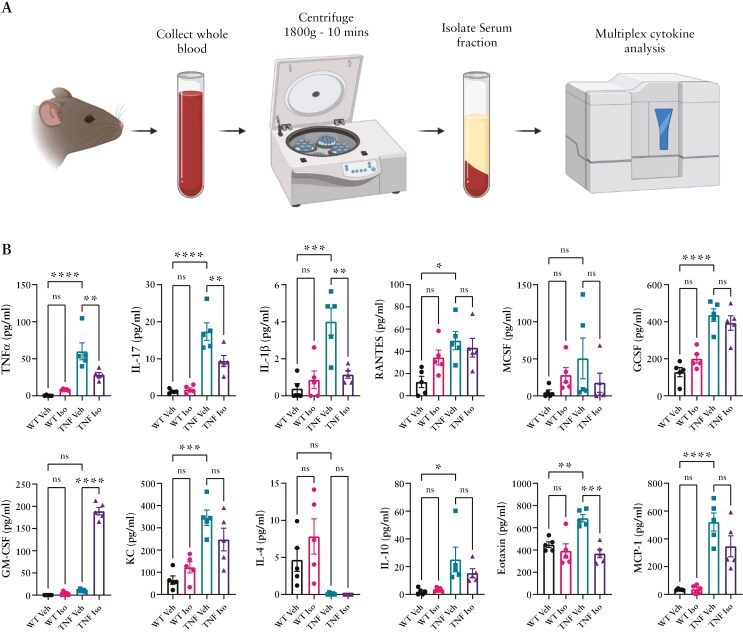
Serum cytokine profile of WT vs TNF^ΔARE^ and the impact of Isoniazid administration. (A) Schematic representation of serum collection and processing prior to multiplex cytokine analysis. (B) Serum cytokine profile of numerous inflammatory cytokines and chemokines. Data are presented as mean ± SEM of *n* = 5 mice per group isolated from 3 separate experimental groups. Statistical analyses were performed using a 1-way ANOVA with Tukey’s post hoc test. *P < .05, **P < .01, ***P < .001, ****P < .0001.

With spleen enlargement significantly reduced in TNF^ΔARE^ mice that received Isoniazid treatment, we sought to further investigate the systemic inflammatory profile of TNF^ΔARE^ mice. Using LUMINEX bead-based multiplex assays, we characterized a comprehensive array of cytokines, chemokines, and growth factors in the serum of untreated or Isoniazid-treated TNF^ΔARE^ mice and compared them to WT litter-matched controls (Figure 3). Analysis demonstrated that untreated TNF^ΔARE^ mice are systemically inflamed with significantly increased serum levels of: TNFα, IL-17, IL-1β, RANTES, GCSF, KC, Eotaxin, IL-10, and MCP-1. TNF^ΔARE^ mice that received Isoniazid, however, had significantly reduced levels of TNFα (*P* < .01), IL-17 (*P* < .01), IL-1β (*P* < .01), and Eotaxin (*P* < .001) compared to their untreated counterparts, with RANTES and KC also trending toward a decrease in abundance. Of note, systemic IL-4 levels were frequently below the detection limit within TNF^ΔARE^ mice with Isoniazid having no effect on its expression. Interestingly, GM-CSF production was significantly elevated in TNF^ΔARE^ mice that received Isoniazid (*P* < .0001), a phenomenon that did not occur, however, within the WT Isoniazid-treated counterparts. These data provide an in-depth insight into the systemic microenvironment of the TNF^ΔARE^ mice, which until this time had not yet been profiled.

### 3.4. Terminal ileal inflammation is reduced with Isoniazid treatment

**Figure 4.**
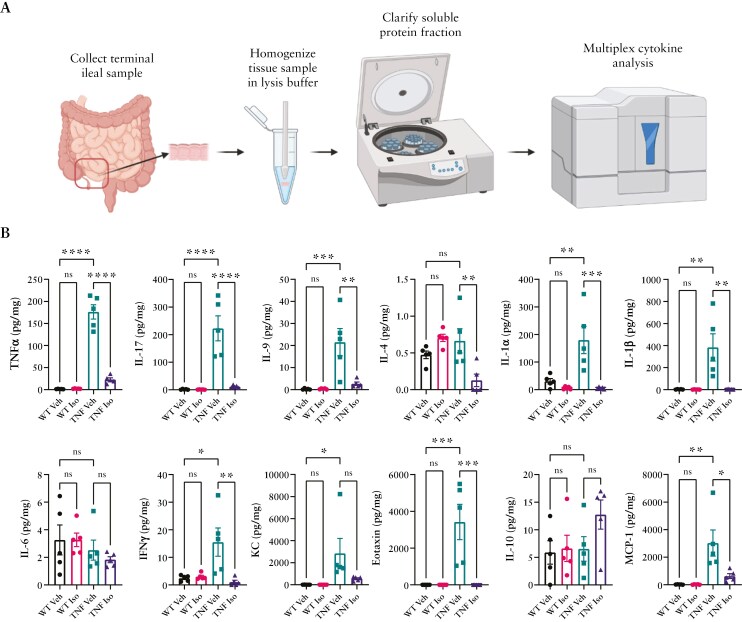
Terminal ileal inflammation profile of WT and TNF^ΔARE^ and the impact of Isoniazid administration. (A) Schematic illustration of serum collection and processing prior to multiplex cytokine analysis. (B) Serum cytokine profile of numerous inflammatory cytokines and chemokines. Data are presented as mean ± SEM of *n* = 5 mice per group isolated from 3 separate experimental groups. Statistical analyses were performed using a 1-way ANOVA with Tukey’s post hoc test. *P < .05, **P < .01, ***P < .001, ****P < .0001.

The positive impact of Isoniazid on systemic inflammation directed us to profile the microenvironment of the terminal ileum (Figure 4). Analysis of ileal tissue homogenates from WT and TNF^ΔARE^ mice found significantly elevated levels of: TNFα, IL-17, IL-9, INFγ, KC, Eotaxin, and MCP-1 in TNF^ΔARE^ mice, indicative of an inflamed tissue. Isoniazid administration for 4 weeks significantly reduced the levels of many of these inflammatory mediators, importantly: TNFα, IL-9, IFNγ, Eotaxin, and MCP-1. Most notably, IL-17, the T-cell-derived cytokine known to be potently driven by the murine commensal SFB,^40^ was almost undetectable after treatment which correlated to a significant reduction in infiltrating CD4^+^RORγt^+^ Th17 cells (Supplementary Figure S2). Further, histological assessment of the ileum highlighted that this reduction in inflammation correlated to a nonsignificant reduction in villus blunting and crypt hyperplasia (Supplementary Figure S3a and b). Additionally, although not significant, there were improvements in the histological inflammatory index with reduced ileal cellular infiltration, epithelial alterations, and submucosal edema in TNF^ΔARE^ mice treated with Isoniazid (Supplementary Figure S2c). These data suggest that in TNF^ΔARE^ mice, Isoniazid may be effective at reducing intestinal inflammation, though whether this effect is through direct action or through modulating host–microbe interactions remained uncertain.

### 3.5. Isoniazid treatment limits the expansion of epithelial-associated SFB within the terminal ileum of TNF^ΔARE^ mice

**Figure 5.**
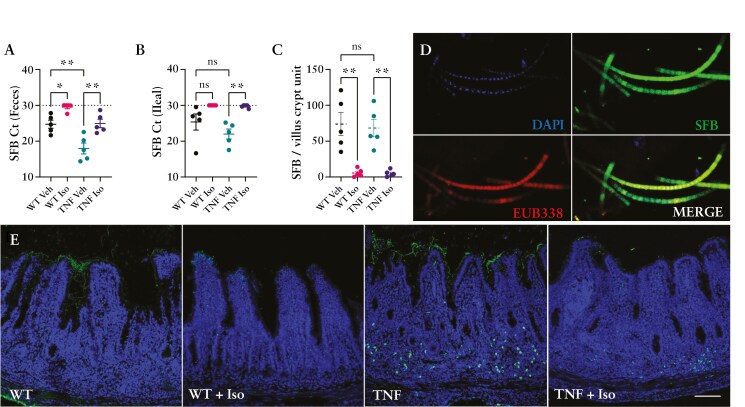
Isoniazid limits the expansion of epithelial-associated Segmented filamentous bacteria within the terminal ileum of TNF^ΔARE^ mice. PCR analysis of the abundance of (A) fecal and (B) ileal–epithelial–adherent SFB (dotted line delineates 30 cycle threshold of nonspecific amplification). (C) Number of SFB filaments per villus–crypt unit averaged from 3 separate 10 µm ileal sections of 12-week-old WT and TNF^ΔARE^ mice treated with water (Veh) or 10 mg/kg/day Isoniazid (Iso). (D and E) Representative FISH of SFB counterstained with the pan-eubacteria probe EUB338 (D – bottom left panel) and imaged within ileal sections from 12-week-old WT and TNF^ΔARE^ mice ± Isoniazid treatment. Scale bar = 500 µm DAPI (D – top left panel), SFB FISH probe (D – top right panel). Data are represented as the mean or mean ± SEM from 5 individual mice per group analyzed from 3 separate treatment experiments. Statistical analyses were performed using a 1-way ANOVA with Tukey’s post hoc test. *P < .05, **P < .01.

With the reduction in intestinal and systemic IL-17, combined with recently published data highlighting the obligatory role for ileal SFB colonization with the manifestation of the TNF^ΔARE^ ileitis,^24^ we sought to determine the impact of Isoniazid on ileal SFB colonization in these mice. Through qPCR assessment, SFB was found to be present within all mice in our colony; however, the quantity of SFB detected was significantly higher in the feces of TNF^ΔARE^ mice (*P* < .01) (Figure 5A). As intestinal inflammation, along with SFB colonization, is restricted heavily to the terminal ileum,^40^ we next collected and analyzed luminally flushed ileal sections to assess the abundance of epithelial-adhered SFB only. Intriguingly, we found no significant difference in the abundance of SFB either by qPCR (Figure 5B) or by FISH immunostaining and counting of SFB filaments per villus–crypt unit (Figure 5C and D). Analysis of SFB within the feces and adhered to the epithelium of WT and TNF^ΔARE^ mice that had received Isoniazid treatment demonstrated a significant reduction in SFB load within the feces (Figure 5A) and almost a complete absence of SFB adhered to the ileal epithelium (Figure 5C and E). This loss of SFB abundance correlates to the reduction in terminal ileal and systemic inflammation we previously noted (Figures 3 and 4).

### 3.6. Isoniazid prophylaxis reduces the formation of mesenteric TLOs which are found to be associated with SFB endospores

**Figure 6.**
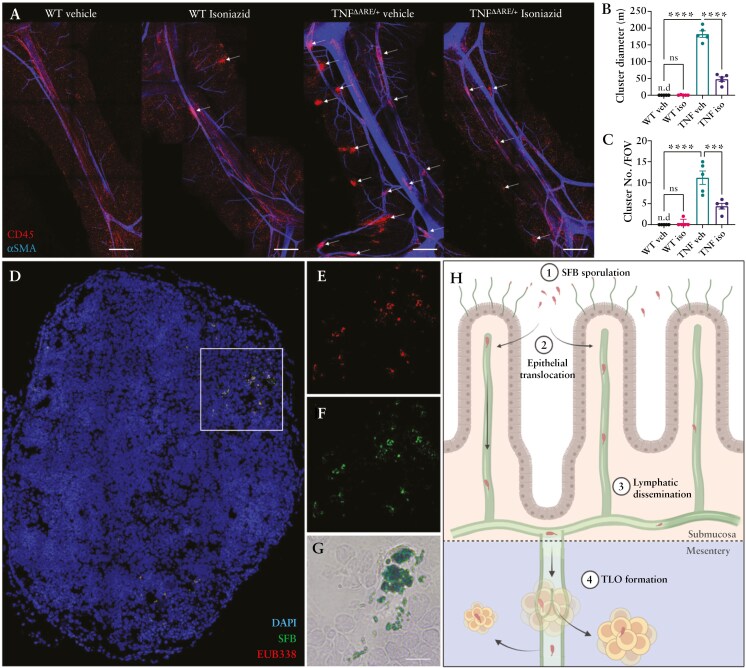
Isoniazid diminishes the progression of TNF^ΔARE^ disease characteristics and extraintestinal mesenteric tertiary lymphoid organ formation. (A) Representative whole-mount immunofluorescent images and quantification of immune cells (CD45—red) within the terminal ileal mesenteric arcade of WT and TNF mice ± Isoniazid treatment with the vascular network (αSMA—blue) Scale bar = 500 µm. (B) CD45 cluster diameter and (C) number per terminal ileal arcade (field of view—FOV). (D) Cryo-sectioned 5-µm sections of fully developed lymphatic associated TLOs from the ileal mesentery of 28-week-old TNF^ΔARE/+^ were stained using (E) the pan-bacterial EUB338 (red—Alexa 647) or (F) SFB-specific (green—Cy3) oligonucleotide FISH probes with DAPI staining all nuclei (blue). A sequential section from the same TLO (G) was stained using the Ward’s Chemistry endospore staining protocol with endospores staining green (malachite green) against a pinkish counterstained background of Safranin-O. Scale bar = 10 µm. (H) Schematic representation of predicted mechanism with (H1) SFB sporulation, (H2) translocation through the intestinal epithelium, (H3) dissemination through the ileal mesenteric lymphatic network, and (H4) TLO formation within the associated ileal mesentery specifically around leaky collecting lymphatic vessels. TLO image is representative of 3 sequential sections taken from 2 to 3 fully encapsulated TLOs isolated from *n* = 5 28-week-old TNF^ΔARE^ mice. Graphical depiction of predicted SFB dissemination mechanism was created using BioRender.

Extraintestinal immune cell clustering within the mesentery, indicative of the formation of disease-associated TLOs,^19,20,41^ was next assessed. Whole-mount immunofluorescent staining of the terminal ileal mesentery of vehicle and Isoniazid treatment WT mice highlighted that CD45^+^ immune cells are evenly distributed throughout the mesentery and associated fat (Figure 6A and Supplementary Figure S1). Within the TNF^ΔARE^ ileal mesentery, associated closely with αSMA^+^ collecting lymphatic vessels, immune cells (CD45^+^) were found abundantly in dense clusters. Notably, these clusters were only present within the terminal ileal arcade, extending from the gut-border mesenteric adipose, along collecting lymphatic vessels, and throughout the clear spaces of the mesothelium (Figure 6A). Unlike at later time points >20 weeks of age, TLOs/immune cell clusters were not macroscopically visible under the dissection microscope due to the presence of opaque mesenteric adipose tissue and required optical clearing combined with immunofluorescent visualization for detection. Ultimately, we found that TNF^ΔARE^ mice that received Isoniazid treatment had an overall reduced abundance of immune cell clustering within mesentery, which were not only reduced in number, but also in size (Figure 6B and C). With SFB having previously been demonstrated to drive the formation of secondary as well as tertiary lymphoid tissue development,^42^ we hypothesized that mesenteric TLOs within the TNF^ΔARE^ may be driven by SFB itself. Using the pan-eubacteria oligonucleotide FISH probe EUB338, we detected an abundance of bacteria within sectioned TLOs isolated from 28-week-old TNF^ΔARE^ mice (Figure 6D and E). Co-staining using the SFB-specific probe^43,44^ demonstrated that a vast majority of the EUB338 positive signal within the TLO was SFB (Figure 6F). Due to their nonfilamentous structure and small size (1-5 µm), we speculated that these bacteria were in fact SFB-derived endospores which was then confirmed using Ward’s chemistry malachite green endospore staining (Figure 6G). Together, these data highlight an abundance of SFB endospores within the TLOs isolated ileal mesentery of TNF^ΔARE^ suggesting that these structures may in part be driven by SFB endospores disseminating from the ileum through the associated mesenteric lymphatic network (illustrated Figure 6H). Though this remains a speculative hypothesis due to experimental limitations, this is supported by previous works that demonstrate SFB’s ability to drive the formation of TLOs in other incidences^42^ and would further explain the restricted formation of these structures to only the mesentery of the SFB-colonizing terminal ileum.^19,20^

### 3.7. Increased SFB abundance within TNF^ΔARE^ mice is correlated with altered IL-22 and AMP production

**Figure 7.**
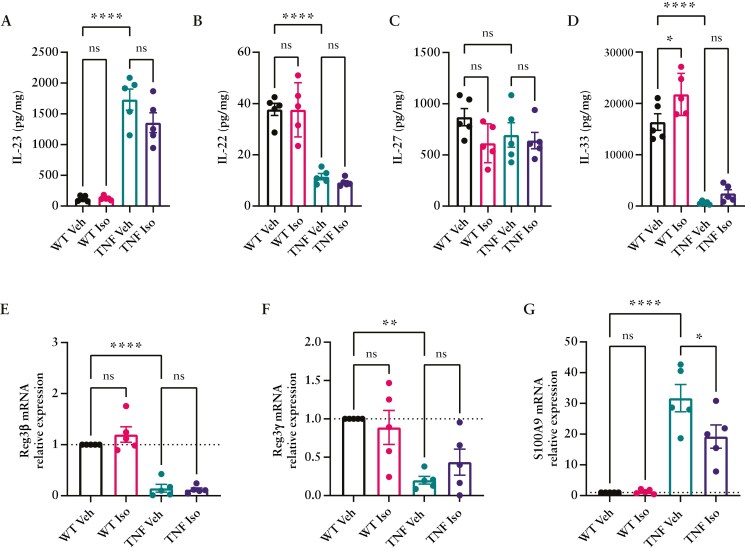
TNF^ΔARE^ mice lack production of IL-22 within the ileum resulting in decreased antimicrobial peptide production. Cytokine evaluation of (A) IL-23, (B) IL-22, (C) IL-27, (D) IL-33 measured by Luminex bead-based multiplexing of ileal tissue homogenates from 12-week-old WT and TNF^ΔARE^ treated for the previous 4 weeks with water (veh) or 10 mg/kg/day Isoniazid (Iso). Measurements of intestinal sections for antimicrobial peptides (E) Reg3β, (F) Reg3γ, and (G) S100a9 were performed using qPCR. Dotted line indicates normalized mRNA expression compared to WT vehicle controls. Data are represented as mean ± SEM of *n* = 5 mice analyzed from 3 separate treatment experiments. Statistical analyses were performed using a 1-way ANOVA with Tukey’s post hoc test. *P < .05, **P < .01, ****P < .0001.

While previous literature had documented the loss of Paneth cells in TNF^ΔARE^ along with reduced AMP production,^23,45^ investigations as to whether the IL-22-dependent mechanism of their production remains intact are yet to be investigated. Furthermore, as IL-22-induced Reg3γ production by Paneth cells is essential in regulating the cyclical adherence of SFB,^25^ we hypothesized that a section of this signaling cascade may be disrupted specifically in TNF^ΔARE^ mice. Thus, to address this hypothesis, we quantified cytokines known to regulate microbiota-driven AMP production within ileal tissue of WT and TNF^ΔARE^ mice as well as those that had received Isoniazid. Further supporting TNF^ΔARE^ as a relevant model of IBD, the Th17-associated cytokine IL-23 was significantly elevated in the TNF^ΔARE^ ileum compared to WT littermates and was unchanged with Isoniazid treatment (Figure 7A). IL-22, induced by IL-23R stimulation of tissue-resident Th17 and ILCs, however, was significantly reduced in TNF^ΔARE^ mice and unaffected by Isoniazid administration (Figure 7B). IL-27, a known inducer of gram-negative targeting AMPs,^46^ was unchanged between groups suggesting some signaling pathways remained unaffected (Figure 7C). IL-33, a pleotropic alarmin, was seen to be significantly reduced in TNF^ΔARE^ ileal samples compared to WT controls and was unaltered by Isoniazid administration (Figure 7D). Finally, qPCR analysis of epithelial-derived AMPs: Reg3β, Reg3γ, as well as neutrophil-derived S100A8/9 (Fecal Calprotectin) highlighted the altered expression of AMP transcripts within the TNF^ΔARE^ ileum with significantly lower transcripts of Reg3β and Reg3γ (Figure 7E and F) and elevated Calprotectin which was significantly reduced during Isoniazid prophylaxis (Figure 7G). These data highlight a possible deficit in the ability for TNF^ΔARE^ mice to produce IL-22 and associated AMPs despite sufficient IL-23 induction, ultimately failing to regulate the cyclical colonization of SFB within the ileum.

### 3.8. TNF^ΔARE^ mice have reduced IL-22 production through increased deadenylase activity

**Figure 8.**
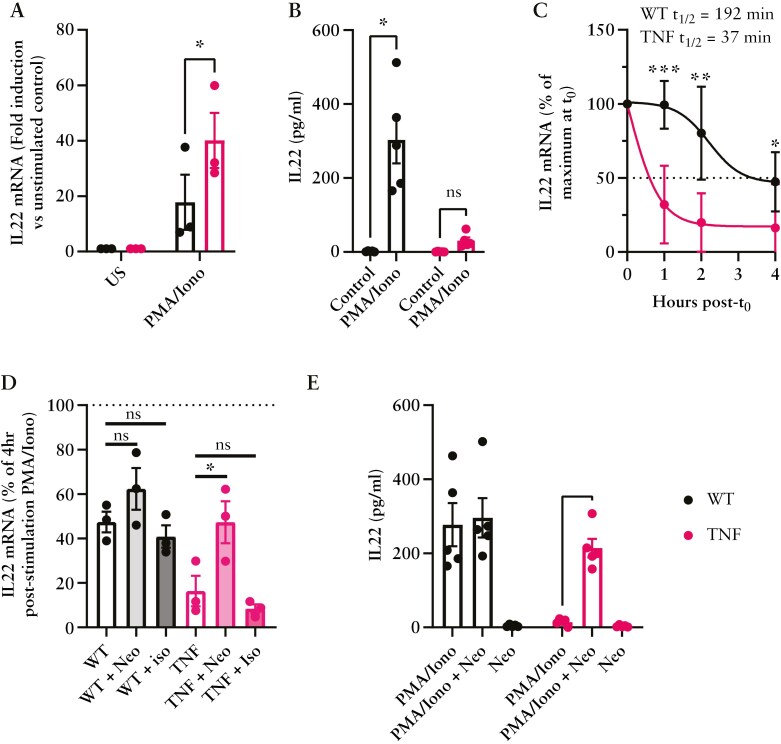
TNF^ΔARE^ mice display altered mRNA degradation in isolated splenocytes. Assessment of mRNA stability in isolated splenocytes from 12-week-old WT and TNF^ΔARE^ through pre-stimulation with PMA (50 ng/mL) + Ionomycin (1 µg/mL) for 4 hours. (A) Quantification of fold induction of IL-22 mRNA within PMA/Ionomycin stimulated splenocytes of WT or TNF^ΔARE^ mice. (B) ELISA quantification of IL-22 produced from sample-matched PMA/Ionomycin stimulated WT and TNF^ΔARE^ splenocytes. (C) The calculated decay of PMA/Ionomycin-induced IL-22 mRNA after administration of Actinomycin-D was measured at indicated hours using qPCR and is expressed as a normalized percentage of the mRNA present at t0 (4 hours post-PMA/ionomycin stimulation). (D) Percent IL-22 mRNA remaining 4 hours post-stimulation with PMA + Ionomycin after co-incubation with deadenylase inhibitor Neomycin (5 µg/mL), or Isoniazid (5 µg/mL). (E) IL-22 protein production from splenocytes measured after 24 hours of PMA + Ionomycin stimulation and the impact of Neomycin treatment (5 µg/mL). Data are represented as mean ± SEM of 3 independent experiments performed on splenocytes isolated from 3 to 5 mice of each genotype. Statistical analysis for both mRNA and protein change was assessed using a 2-way ANOVA with Kruskal–Wallis post hoc test. mRNA decay determined using a nonlinear regression model log (agonist) vs response (3 parameters) to calculate the t_1/2_ or EC50 of IL-22 mRNA. *P < .05, **P < .01, ***P < .001.

The mutation of the 3′UTR of the TNF transcript prevents the posttranscriptional degradation of TNF mRNA by zinc-finger proteins with RNA-binding properties including TTP.^15,17^ With recent evidence highlighting the presence of ARE elements within the 3′UTR of other gene transcripts, we hypothesized that TTP may be influencing the production of other essential intestinal proteins.^47^ We therefore sought to assess the stability of IL-22 production, an essential IL-23-driven cytokine involved in the regulation of intestinal microbiota homeostasis through induction of AMP secretion by Paneth cells, and whose mRNA degradation has recently been demonstrated to be directly regulated by TTP.^43,48^ With its significant downregulation previously noted within the inflamed ileum of TNF^ΔARE^ mice (Figure 7B), it gave credence to our hypothesis. To address the mRNA dynamics of IL-22 production, splenocytes from WT and TNF^ΔARE^ 12-week-old mice were isolated and stimulated in vitro. The initial induction of IL-22 mRNA was measured via qPCR after 4 hours of stimulation with PMA and Ionomycin and was found not only to be induced, but significantly increased in TNF^ΔARE^ mice (*P* < .05, Figure 8A). However, assessment of IL-22 protein production from TNF^ΔARE^ cells stimulated with PMA and Ionomycin for 24 hours was significantly reduced compared to the induction seen in splenocytes from WT counterparts (Figure 8B). Due to the previously documented TTP-dependent regulation of IL-22 mRNA stability, we then assessed whether mRNA decay was altered in TNF^ΔARE^ splenocytes. Therefore, after 4 hours of stimulation with PMA + Ionomycin, transcriptional activity was halted using the RNA-polymerase inhibitor Actinomycin-D (5 µg/mL), and mRNA stability was quantified over a 4-hour period. Data shows the calculated half-life (t_1/2_) of IL-22 mRNA to be 192 minutes in WT splenocytes while only 37 minutes in those derived from TNF^ΔARE^ (*P* < .001 at 1 hour, Figure 8C). While no selective inhibitor currently exists for TTP, preincubation of splenocytes with Neomycin, a well-described nonselective deadenylase inhibitor,^49,50^ significantly improved the relative abundance of TNF^ΔARE^ splenocyte IL-22 mRNA 4 hours post-stimulation (Figure 7D), partially restoring IL-22 protein production from those cells (Figure 8B). Isoniazid, used in the same manner as Neomycin, had no notable impact on levels of IL-22 transcript (Figure 8C) or protein (Figure 8D). Together, these data highlight that through the genetic stabilization of the TNF transcript through 3′UTR manipulation, other ARE-containing mRNAs are preferentially degraded here epitomized by the loss of inducible IL-22 protein production.

## 4. Discussion

Stimulation of immune cells within the lamina propria due to increased epithelial permeability is known to play a critical role in the chronicity of inflammation in CD. Whether through the release of damage-associated molecular patterns during tissue destruction, or increased microbial dissemination, the transmural inflammation associated with the disease is viewed as a prominent target for therapy.^51^ Furthermore, the figurative explosion in microbiome studies has driven research into a better understanding of the pathological role of infectious and commensal intestinal microbes as drivers of disease and provided a plethora of targets for therapeutic intervention.^52,53^ Due to similarities of microbial pathologies, the exact bugs proposed to initiate and drive IBD are contentious. No more is this epitomized than in the speculative role of mycobacterial infection in IBD. Debated for more than 2 decades, the role of mycobacteria in the pathogenesis of IBD has been met with fierce debate and confusion intensified by cellular and structural similarities observed in macroscopic lesions associated with mycobacterial infections^54^ and IBD.^55,56^ Additionally, many of the treatments used broadly within the clinical setting have direct and indirect impacts on the host microbiome including antibiotics such as Isoniazid, a commonly used prophylactic measure to combat the resurgence of latent TB prior to anti-TNFα treatment of IBD.^57^ Despite this clinical relevance, however, no significant literature exists determining whether Isoniazid has a therapeutic benefit alone thus spurring this investigation. With latent TB thought to be present in ¼ of the global population, Isoniazid is a critical medication in the global and endemic control of active TB cases, as well as its combinatorial use in the prevention of latent TB reactivation. Along with the increasing global burden of TB as well as IBD, understanding the pathogenesis and treatment of these 2 diseases is of utmost importance. Here, we have identified that Isoniazid alone is able to successfully limit the development of terminal ileitis in a human-relevant mouse model of CD through modulation of host–microbe interactions of the previously described mouse pathobiont, SFB. We also highlight a previously undescribed deficit in the ability for TNF^ΔARE^ mice to produce IL-22 in response to stimulation due to increased degradation of its mRNA. These data present novel therapeutic pathways to be explored in CD, though more work must be done to fully elucidate its impact on the host and/or intestinal microbes.

### 4.1. Host-directed anti-inflammatory impact of Isoniazid

Since its discovery in the early 1950s, Isoniazid has demonstrated numerous TB-independent actions including early descriptions by Swiss TB physicians on its mood-elevating impact on patients.^58,59^ In the 70 years since its first clinical use, numerous groups have described immunomodulatory actions of Isoniazid independent of its proposed antimycobacterial function. This includes inflammatory-associated immune cells such as neutrophils, which were seen to be able to metabolize Isoniazid into its NAD^+^ adduct through the action of MPO, disturbing cell metabolism, reducing the overall inflammatory profile of the cell.^60^ While we found that Isoniazid did not alter tissue MPO activity in our system, numerous pro-inflammatory cytokines were significantly reduced suggesting it promotes anti-inflammatory responses. These data, combined with evidence demonstrating that MPO activity and abundance are not readily correlative in rodent models of IBD,^61^ highlight that using MPO activity as an ultimate marker of intestinal inflammation is unreliable and should be supported by additional analyses. Debatably more dramatic than altering neutrophil metabolism, Isoniazid treatment of TB-infected mice has been shown to drive apoptosis of activated T cells in vivo which, while promoting clearance of TB infection, supresses host immunity and inflammatory cytokine production acutely, enhancing the overall chance of reinfection.^62^ The significant loss of T-cell-associated cytokines, TNFα, IL-17, and IL-9 with Isoniazid treatment, suggests that the reduced inflammatory state of the TNF^ΔARE^ mice may be attributed to such mycobacterial-independent mechanisms although require future validation. Further, we propose that the levels of MPO remaining within the ileum may act as a catalyst for Isoniazid metabolism into NAD^+^ adducts, reducing inflammation locally similarly to previously described mechanisms of neutrophil metabolic reprogramming.^60^ Overall, Isoniazid may present a novel anti-inflammatory therapy for the treatment of IBD, one whose mechanisms are likely scarcely known but show promise.^63^

### 4.2. Microbiome-directed actions of Isoniazid

The complex interaction between the microbiome and its host has recently begun to be probed in great detail, but with a majority of the focus restricted to that of the highly abundant and influential bacterial compartment. What is known, however, is that immunomodulatory therapies, as well as the high global use of antibiotics, impact this compartment heavily and can have distinct impacts on the progression of numerous diseases.^64–66^ Moreover, the use of antibiotics as a possible therapeutic agent in the treatment of IBD continues to garner much attention having been shown to be effective for the induction of remission and in the postoperative management of patients undergoing IBD-related surgery.^64,67,68^ While there is increasing evidence supporting the essential role of the intestinal microbiome in the pathogenesis of IBD, noninfectious commensal microbes (pathobionts) identified to drive disease remain scarce.^69^ The mouse commensal, SFB was recently identified as the essential driver of the IL-17 dominant CD-like terminal ileal inflammation within the TNF^ΔARE^ mouse.^24^ Though Metwaly and colleagues propose the existence of other intestinal microbes that may drive a similar response from this and other mouse models of IBD, they currently remain unidentified and moreover are likely host-species-specific. In our investigation, we observed that the fecal load of SFB was significantly elevated within TNF^ΔARE^ mice compared to WT counterparts but the abundance of epithelial-associated SFB appeared indifferent suggesting something aside from simple overgrowth. SFB-derived endospores, or initiating offspring, are produced naturally as a part of the life cycle of SFB, rather than a stress response to nutrient limitation.^70^ These endospores are highly infectious in nature, with daughter cells promoting host–host transmission and continual colonization of the original host. One of the hardiest forms of life, bacterial endospores are highly resistant to immunological reactive oxygen and nitrogen species,^71^ lysosomal degradation,^72^ and surviving environmental stresses like desiccation, heat, and UV.^73^ Therefore, we propose that not only is it likely that these identified SFB endospores are contributing to the chronic inflammation found within the TNF^ΔARE^ mice, but we suggest are directly involved in the formation of mesenteric TLOs. This granulomatous response to a non-resolvable infection driving local inflammation which is also likely dependent on the host susceptibility of the model used. Whether human-specific pathobionts similarly drive this type of response, however, remains unknown but under investigation with our current working hypothesis. We speculate that there is enhanced and perhaps unregulated, sporulation of SFB within TNF^ΔARE^ mice and these stress-induced endospores pass through the “leaky” gut, ultimately driving the formation of mesenteric TLOs in a similar manner as previously shown.^42^ With recent evidence demonstrating the requirement of autocrine TNFα/TNFR1 signaling to control both cell differentiation and intestinal mucin homeostasis,^74^ we suggest that this ileal leakiness is likely not pathologically induced but rather a spontaneous event amplified by SFB colonization. This TNFα/TNFR1-dependent permeability hypothesis is corroborated by previous works highlighting the role of intestinal epithelial-specific TNFα (TNF^i∆ARE/i∆ARE^)^75^ and the neutralization studies of sTNFα or TNFR1.^15,18,20^

While we demonstrate that Isoniazid administration resulted in the dramatic loss of epithelial–adherent SFB, due to the continued resistance to in vitro culture, we are currently unable to delineate whether Isoniazid has a direct impact on SFB through bactericidal actions. Additionally, while the mono-colonized SFB mice have allowed the development of an in vitro coculture system,^76^ this too would fail to allow us to differentiate between the host-directed and SFB-directed activities of Isoniazid. Perhaps most importantly, global intestinal dysbiosis has been observed within the TNF^ΔARE^ mice^22,23^ and thus, these SFB-independent microorganisms may be also affected by Isoniazid treatment including resident, yet to be fully characterized, non-tuberculoid mycobacterium. Overall, while we can conclude that Isoniazid has therapeutic potential in the treatment of terminal ileitis in the TNF^ΔARE^ model, the underlying mechanism of its action remains incomplete and is thus under continued investigation.

### 4.3. Disturbed cytokine production in TNF^ΔARE^ mice

Despite being created more than 25 years ago, the TNF^ΔARE^ mouse model of terminal ileitis still provides a human-relevant disease model of IBD. Through deletion of the 3′UTR of the TNF transcript, mRNA stability is greatly improved resulting in increased TNFα protein production.^15^ Research on RNA-binding protein TTP and the generation of the TTP^−/−^ mouse, highlighted a connection between TNF and TTP that if disturbed, resulted in strikingly similar phenotypes of cachexia, arthritis, and intestinal inflammation highlighting the importance of TNFα and its regulation in arthritis and IBD pathogenesis.^15,17,47,77,78^ As such TNFα and TTP have been prominent targets for therapeutic intervention.^79–82^ Our data demonstrate for the first time that other ARE-containing transcripts are disproportionately degraded in TNF^ΔARE^ mice. IL-22, an essential cytokine in the production of AMPs, is greatly reduced in the TNF^ΔARE^ intestine through the increased degradation of its mRNA via excess deadenylase activity, likely TTP-mediated.^48^ Whether this mechanism presents an avenue for therapeutic intervention either through exogenous IL-22 supplementation or TTP inhibition remains to be investigated.^83^ Furthermore, whether other ARE-containing transcripts are differentially decayed within the TNF^ΔARE^ host is currently under investigation.

## 5. Conclusion: Isoniazid, more than just a TB-directed antibiotic

Isoniazid, while a potent antimicrobial agent, has numerous documented off-target effects common to small-molecule inhibitors and biologics alike.^84,85^ While unintended, these actions, once studied and understood, often lead to repurposing of drugs or speculations surrounding their off-label usage. Isoniazid has recently celebrated its 70th year of continued use for the treatment of TB; however, since its discovery, numerous TB-independent actions have been uncovered. The interaction of Isoniazid and its metabolized products with the host, their antimicrobial actions, and anti-inflammatory actions have been demonstrated in numerous cases suggesting there may be more to be unearthed. Here, we have highlighted Isoniazid’s therapeutic potential in managing pathobiont-associated intestinal inflammation as well as identifying new underlying mechanisms of disease progression in a human-relevant model of CD.

## Supplementary Material

jjaf032_suppl_Supplementary_Figures_S1-S3

jjaf032_suppl_Supplementary_Figures_Captions

## Data Availability

All data are represented within this manuscript.

## Figure illustrations

All figure illustrations were generated using BioRender.
