# Supplementary figures and images for: Uncovering the therapeutic potential of anti-tuberculoid agent Isoniazid in a model of microbial-driven Crohn’s disease
Source: J Crohns Colitis. 2025 Feb 23;19(3):jjaf032. doi: 10.1093/ecco-jcc/jjaf032 (PMC11920797; doi:10.1093/ecco-jcc/jjaf032)

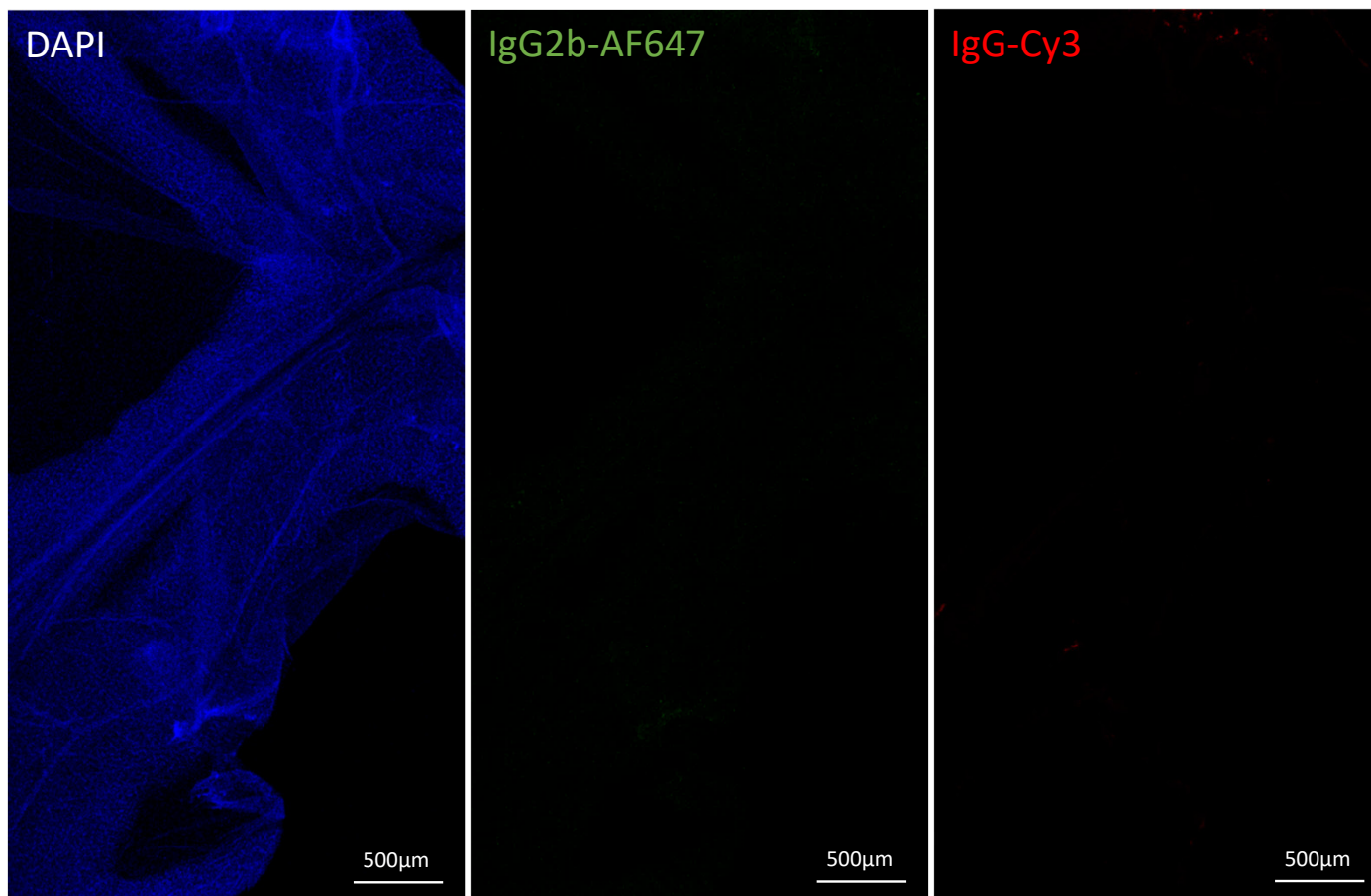

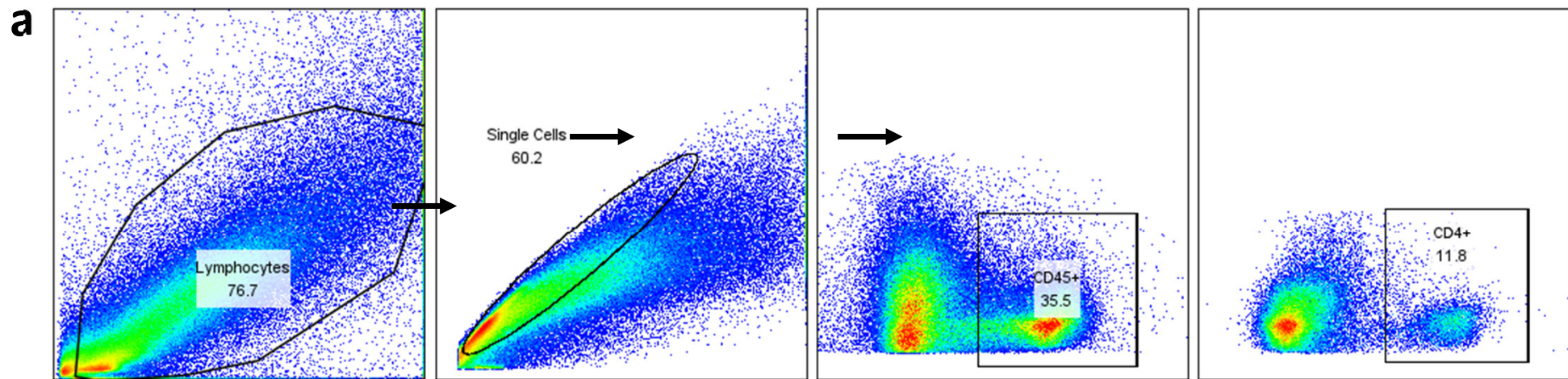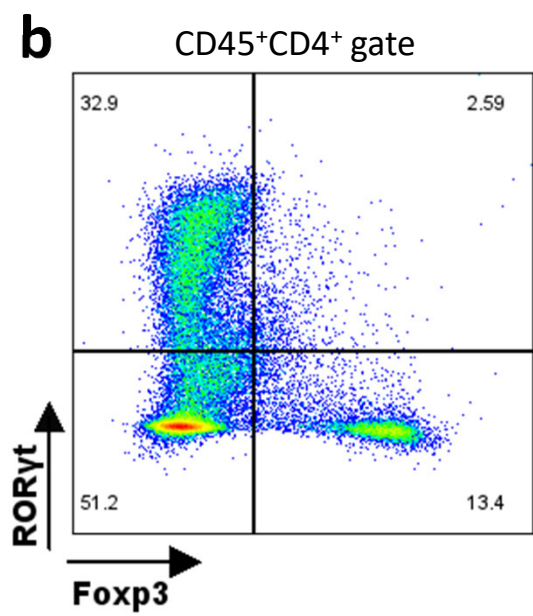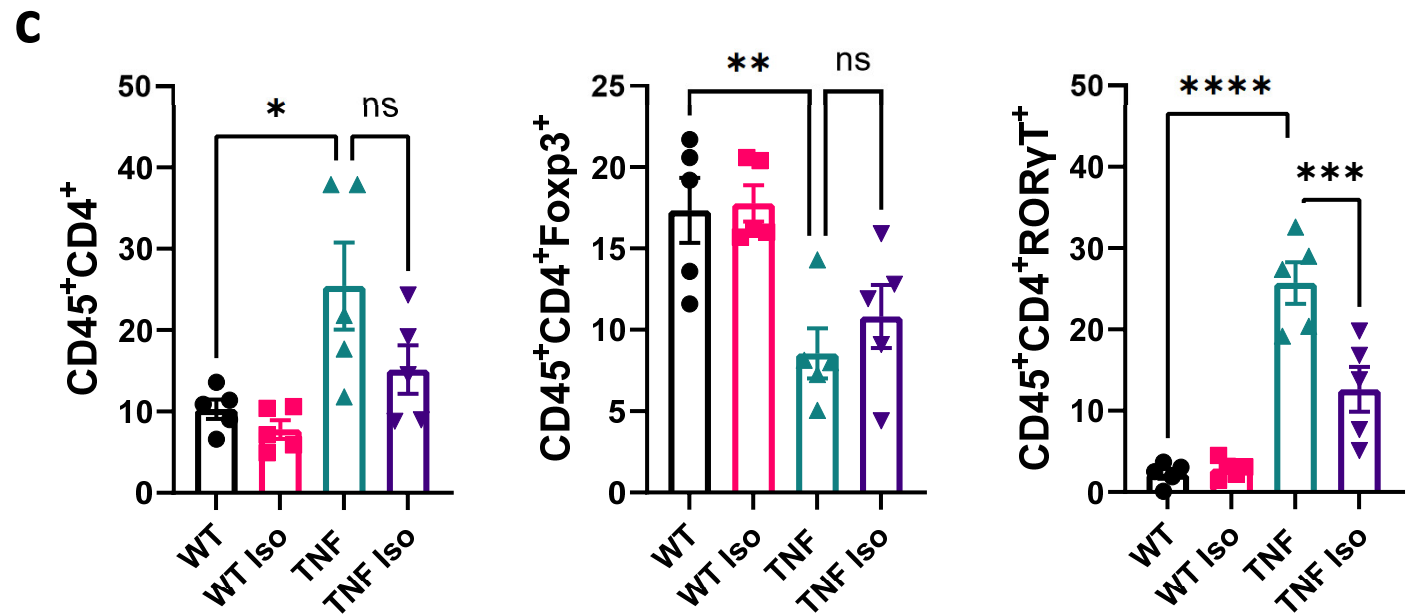

**a**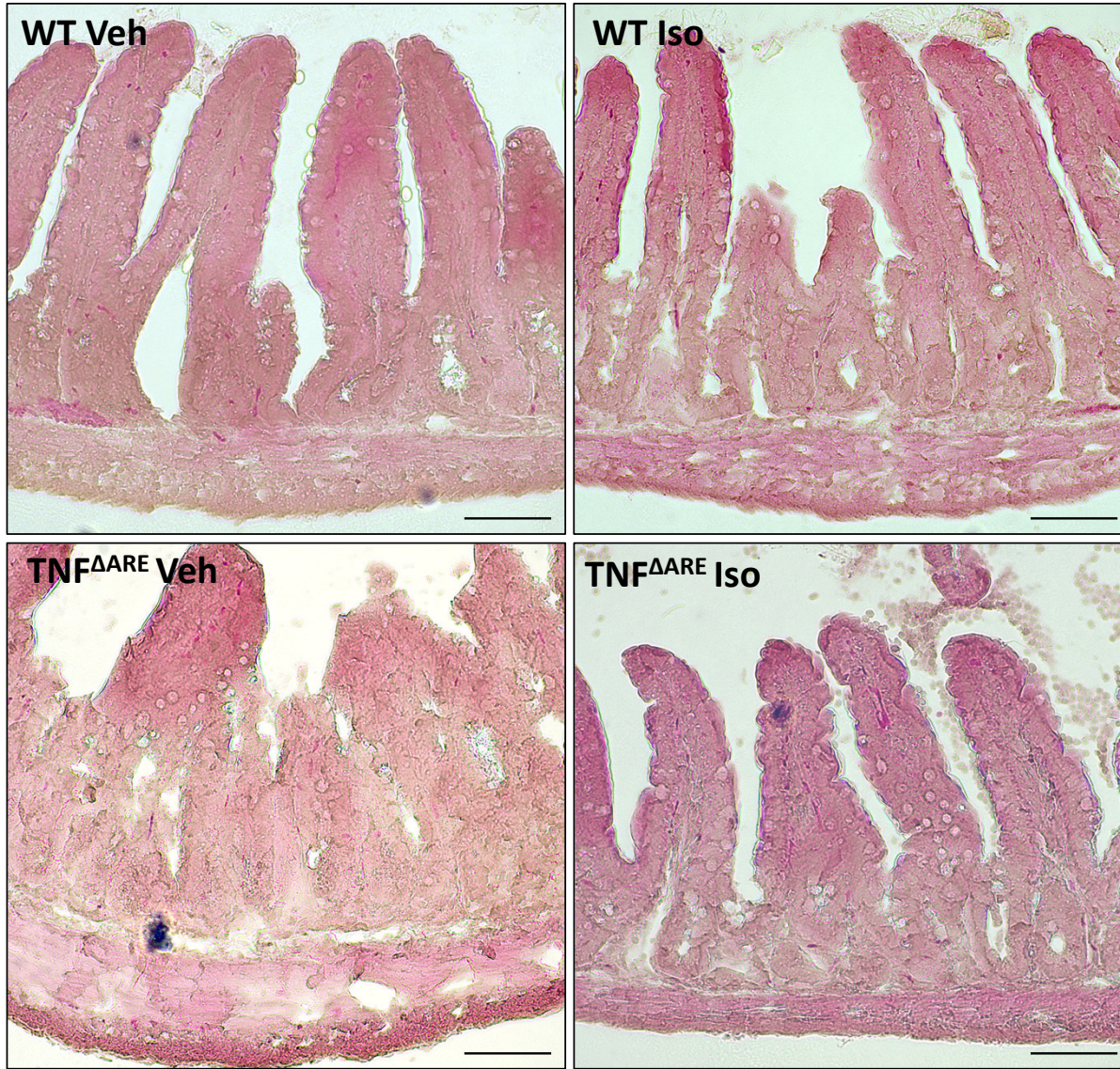**b**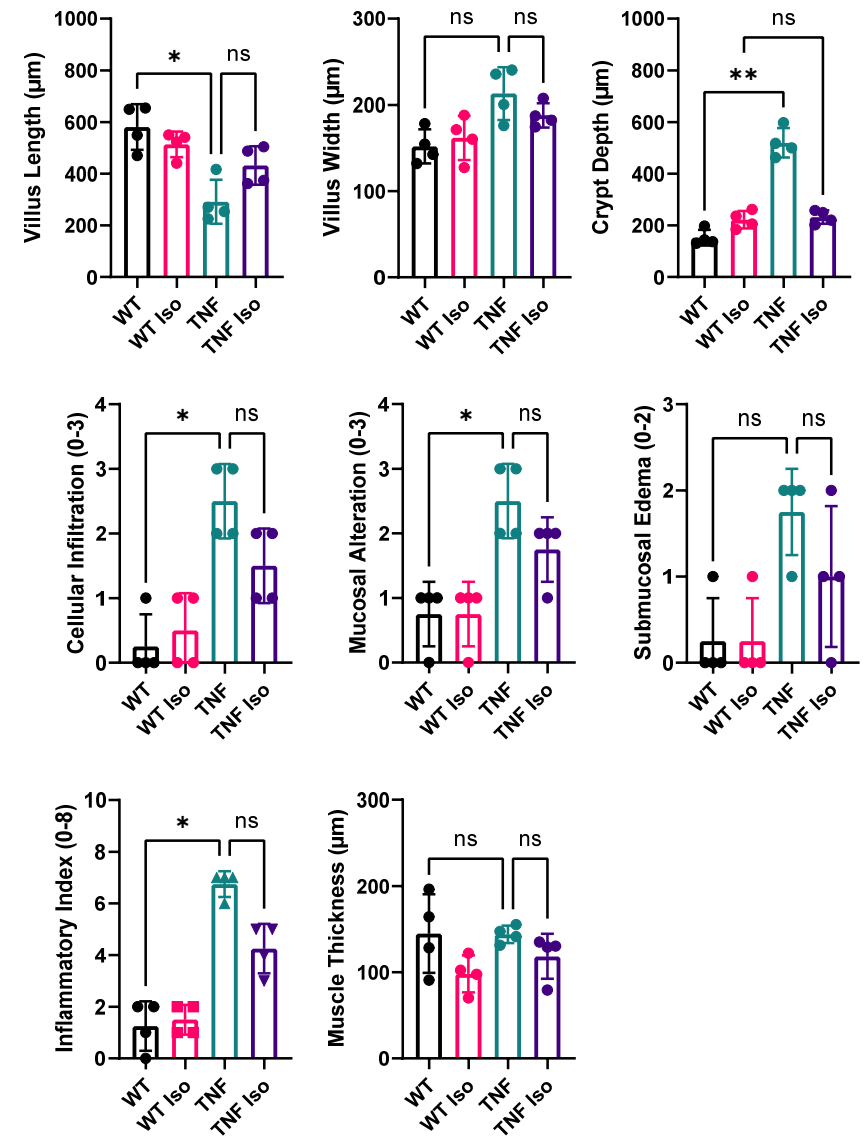

Supplement: jjaf032_suppl_Supplementary_Figures_S1-S3 [file jjaf032_suppl_supplementary_figures_s1-s3.pdf]
