# Supplementary material for: Uncovering the therapeutic potential of anti-tuberculoid agent Isoniazid in a model of microbial-driven Crohn’s disease
Source: J Crohns Colitis. 2025 Feb 23;19(3):jjaf032. doi: 10.1093/ecco-jcc/jjaf032 (PMC11920797; doi:10.1093/ecco-jcc/jjaf032)
Supplement: jjaf032_suppl_Supplementary_Figures_Captions [file jjaf032_suppl_supplementary_figures_captions.docx]

**Supplemental Figure 1:** Representative images of Isotype antibody controls used in whole-mount immunofluorescent imaging of the murine terminal ileal mesentery. DAPI used as a nuclear positive control for orientation and tissue imaging clarity with anti-mouse IgG2b with anti-rat AF647 (Control for anti-mCD45 used in figure 6) and anti-mouse IgG-Cy3 (Control for anti-mαSMA used in figure 6). Scale bar is 500µm.

**Supplemental Figure 2:** Flow cytometry analysis of ileal T-cell subsets. **a)** Gating strategy example performed on single cell suspension isolated from the terminal ileum of mice depicting: Lymphocyte gate, single cell gate, CD45^+^ fraction, CD4^+^ and finally CD4^+^ RORγt^+^ or Foxp3^+^ subsets. Abundance represented as % of **b)** CD45^+^ CD4^+^ T-helper cells, **c)** CD45^+^CD4^+^Foxp3^+^ Treg and **d)** CD45^+^CD4^+^RORγt^+^ Th17 subsets. Data are presented as mean ± SEM of n=5 mice per group performed from at least 4 separate experiments. Data were analyzed using an Ordinary One-way ANOVA with Holm-Sidak’s multiple comparisons test with *P<0.05, **P<0.01, ***P<0.001, ****P<0.0001: ns = not significant.

**Supplemental Figure 3:** Representative Hematoxylin & Eosin staining of **a)** 12-week-old WT or TNF^ΔARE^ ileal sections that received Vehicle (water) or Isoniazid (10mg/kg/day) treatment from 8-weeks-old. **b)** Villus length measure from villus tip to crypt, Villus width measured from mid-length, Crypt depth measured from muscle layer to crypt base, Muscle thickness measured from crypt base to outer muscle layer. **c)** Inflammation characterization including; cellular infiltration, mucosal alterations and submucosal edema totalled into an Inflammatory Index. Data were analyzed using a Kruskal-Wallis nonparametric test with Dunn’s posthoc test based on the variance of the same and are expressed as means ±SD. n=4-5. *P<0.05, **P<0.01.
